# Bibliographical

**Published:** 1892-06

**Authors:** 


### Bibliographical.

A Treatise on Dental Jurisprudence for Dentists and Lawyers, embracing the following subjects: Dental Jurisprudence—Dental Expert Testimony—Identification by Means of the Teeth—Dental Malpractice—Cocaine Poisoning—Fracture of Maxilla during the Extraction of Teeth—Injuries and Death during Anæsthesia—The Jurisprudence of Dental Patents, etc., etc. By Wm. H. Redfuss, D.D.S., author of "Dental Massage," member of the Odontological Society of Pennsylvania, New Jersey State Dental Society, Dental Protective Association of U. S. A. Published by the Wilmington Manufacturing Co., Philadelphia, Pa.

This is a work that has been greatly needed in the dental profession. This subject has nowhere before this had an extended treatment; the only thing like an exception to this statement is the brochure upon this subject in the "American System of Dentistry."

In the preface the author says: "The purpose of this volume is to supply the dental and legal professions with a comprehensive treatise or text-book, covering the subject of Dental Jurisprudence, showing the relations the dental practitioner sustains to the law. It is true that the general principles of Medical Jurisprudence apply equally as well to the dental practitioner, but there are certain special questions which arise exclusively in dental practice, undoubtedly creating a field for a distinct and separate jurisprudence for dentistry. The object of this work is to produce a book of refereuce for technical points of a dento-legal character, enabling those who are desirous of obtaining information or quoting opinions or decisions relative to any dento-legal question, to do so without extensive research."

All the principal leading points of a legal character pertaining to the practice of dentistry are definitely considered in detail, the preparation of which has involved necessarily great labor on the part of the author, as he has drawn from every practicable source for the presentation of the various subjects.

There is an appendix of 250 pages, giving a history of dental legislation, and also every dental law that has ever been enacted in this and foreign countries prior to the present year. This alone makes the work very valuable to every dentist, and it should by all means be in the possession of every practising dentist in the country. It may be obtained of the Wilmington Dental Manufacturing Co., Philadelphia, Pa., or through booksellers generally.

